# Association of HCV Prior Infection and Unprotected Sex on Subsequent HIV Acquisition Risk in the Era of Treatment as Prevention

**DOI:** 10.3389/fmed.2022.902271

**Published:** 2022-05-24

**Authors:** Fangfang Chen, Houlin Tang, Juan Han, Dongmin Li, Peilong Li, Ning Wang, Mengjie Han, Lan Wang, Lu Wang

**Affiliations:** ^1^National Center for AIDS/STD Control and Prevention, Chinese Center for Disease Control and Prevention, Beijing, China; ^2^Department of AIDS/STD Control and Prevention, Zhumadian Center for Disease Control and Prevention, Zhumadian, Henan, China; ^3^Editorial Department of Chinese Journal of Epidemiology, National Institute for Communicable Disease Control and Prevention, Chinese Center for Disease Control and Prevention, Beijing, China

**Keywords:** HCV, unprotected sex, HIV, HIV treatment as prevention, synergistic effect

## Abstract

**Backgrounds:**

Little was known about the impact of HCV prior infection on HIV transmission and acquisition. We aimed to explore whether HCV prior infection and its interactions with unprotected sex affected HIV acquisition.

**Methods:**

This study was conducted among HIV heterosexual serodiscordant couples whose index cases were receiving treatment during 2008–2014 in Zhumadian. At baseline, we collected information on demographics and medical history of ART use, CD4 count, and HIV viral load for index partners, and also HIV and HCV status for non-index partners. For each year's visit, we followed up on sexual behaviors among couples in the recent year and HIV seroconversion of non-index partners. Analyses of the Cox model and synergistic interaction were performed.

**Results:**

We identified 81 HIV seroconversions over 18,370.39 person-years, with the overall HIV seroconversion rate of 0.44 per 100 person-years. Couples, whose index cases were aged 50 years and above, had a baseline viral load >400 copies per ml and no AIDS-defining illness, and newly-initiated ART in the study period had a higher risk of HIV seroconversion. Unprotected sex and HCV prior infection showed a synergistic association with HIV acquisition risk (RERI = 3.65, SI = 0.48, AP = 2.24).

**Conclusion:**

Unprotected sex and HCV infection were independent risk factors associated with HIV acquisition. The coexistence of them might have a synergistic effect on the risk which needs further research.

## Introduction

Human immunodeficiency virus (HIV) and hepatitis C virus (HCV) are both great contributors to the global disease burden. In the absence of an effective vaccine against HIV and HCV, 2.3 million people are coinfected with them (1). The coinfection of HIV and HCV is generally attributed to their shared routes of transmission, such as needle sharing, blood contact, multiple sexual partners, and mother-to-child transmission (2). Among HIV-infected individuals, the highest prevalence of anti-HCV was in injecting drug users (82.4%), followed by men who have sex with men (6.4%) and pregnant women (4%) (3). The primary mode of HCV transmission is highly variable across countries. In resource-limited countries, most HCV infections occur due to the transfusion of unscreened blood and blood products and unsafe healthcare injections. In high-income and many upper middle-income countries, the most common mode of transmission is through injecting drug use (4). The interaction between HIV and HCV coinfection affects the natural history of HCV infection (5). Patients with HIV/HCV coinfection have an accelerated course of HCV disease. Liver diseases are the leading cause of non-AIDS-related deaths in the population with HIV and HCV coinfection. For HCV transmission and acquisition, HIV infection is regarded as an independent risk factor (6). Compared with HCV monoinfections, HIV infections are less likely to spontaneously clear HCV, and their HCV RNA set point tends to be higher, which makes them more infectious to their partners (7). By contrast, little was known about the impact of HCV infection on HIV transmission and acquisition.

HIV prevention has entered a new era with the scientific evidence of “Treatment as Prevention” among serodiscordant couples (where one partner is initially HIV positive— “index case” —and the other HIV negative— “non-index partner”) in clinical trials (8) and real-world evidence (9). Antiretroviral therapy (ART) is rapidly rolling out and becomes the gold standard for the treatment of HIV patients as well as the prevention strategy of HIV transmission. Nevertheless, around 1.5 million [1.0 million−2.0 million] were newly infected with HIV globally in 2020, largely attributed to sexual contact (10). In China, sexual transmission is currently the predominant driver of the recent HIV epidemic as well. The proportion of HIV infections through heterosexual and homosexual contact has steadily increased from 48.3% and 9.1% in 2009 to 71.5% and 23.3% in 2018. The number of new infections among HIV serodiscordant couples has also increased, although HIV transmission has been greatly reduced with the scale-up of ART (11, 12).

Previous studies have reported that consistent condom use was imperfect in couples (13). Unprotected sex is still a great challenge in preventing sexually transmitted HIV. The coexistence of unprotected sex and other sexually transmitted infections (STIs) might amplify the risk of HIV transmission by increasing HIV shedding and lowering the threshold for acquisition (14). Even in circumstances of adequate ART use and serum viral load suppression, HIV transmission may also be enhanced if having both unprotected sex and concurrent STIs (15). STIs can increase the infectiousness of people living with HIV by increasing viral concentration in genital secretions and changing the viral phenotype of HIV variants that favor transmission and facilitate the risk of HIV acquisition in HIV-negative individuals through ulcerative diseases or mucosal inflammation (16). The impact of STIs in increasing both transmission and acquisition vulnerability of HIV has been documented, however, there is no literature addressing whether HCV infection can accelerate the risk of HIV transmission and acquisition as STIs do.

In this study, we explored whether HCV prior infection affected HIV acquisition of non-index partners among HIV serodiscordant couples with index cases receiving ART. We assessed the potential interactions of HCV infection and unprotected sex on HIV acquisition by utilizing data from the cohort of HIV heterosexual serodiscordant couples during 2008–2014 in Zhumadian, China.

## Methods

This study enrolled all HIV serodiscordant couples in Zhumadian of China during 2008–2014, with most index cases infected through contaminated blood and plasma selling practices prevalent in the mid-1990s (17). All participants should be aged 16 years and above (the age of legal consent in China), resident locally for more than 1 year, in a steady sexual relationship with their cohabiting couples, and willing to provide informed consent at study entry.

Eligible couples were given a separate, face-to-face private interview each year by trained staff from the local center for disease control (CDC). At baseline, information on demographics and medical history of ART use, CD4 count, and HIV viral load were recorded for index partners, while HIV and HCV status were collected for non-index partners. For each year's visit, we followed up sexual behaviors among couples in the recent year and HIV seroconversion of non-index partners. Those who were initially confirmed positive for HIV or HCV would be referred to medical institutions for treatment and care. Details of HIV testing can be found in the previous publication (18), including two enzyme-linked immunosorbent assays (ELISA-1, Lizhu, Zhuhai, Guangdong province; ELISA-2, Xinchuang, Xiamen, Fujian Province) and one Western blot assay (WB, Ou'ya, Hangzhou, Zhejiang Province). HCV antibody screening was done through two enzyme-linked immunosorbent assays (ELISA-1, Xinchuang, Xiamen, Fujian Province; ELISA-2, Kehua, Shanghai). All laboratory tests were carried out by the local CDC.

**Figure 1:**
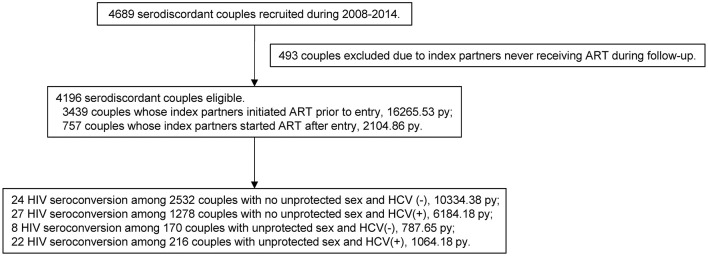
Study profile.

During 2008 and 2014, a total of 4,689 heterosexual couples with at least two visits were included. Due to the high ART coverage, we restricted the analysis to 4,196 couples whose index cases initiated ART before study entry or at the time of baseline (Figure 1).

The primary outcome was HIV seroconversion of the non-index partners during the study period. The date of HIV seroconversion was defined as the midpoint between the date of last HIV-negative test and the date of first HIV-positive test for non-index partners. To accurately estimate the time of HIV seroconversion, we further validated the test results and dates with those in the national HIV surveillance database, a real-time, web-based system that recorded all newly diagnosed HIV cases by medical institutions across the country. Once the two sets of results differed, information from the surveillance system was given priority. The time of follow-up, as recorded in years, started on the date of study entry and ended on the date of HIV seroconversion, data of last HIV-negative visit for non-index partners, or 31 December 2014, whichever came first.

We defined unprotected sex in the recent year within couples to be inconsistent condom use during sex in the recent year, focusing on the impact of 100% condom use on HIV transmission during sexual behavior (17, 19).

### Data Analysis

HIV seroconversion rate was calculated as the number of HIV seroconversion divided by person-years (PY) at risk in 2008–2014. Cox proportional hazards models were used to assess the associations between potential risk factors and HIV seroconversion. The indicators included sex (male vs. female), age (<50 years vs. ≥ 50 years), education (no schooling or primary school, middle school and above), occupation (farmer vs. others), route of HIV infection (blood or plasma donation, blood transfusion, sexual contact, others), baseline CD4 cell count (<200 cells per μL, 200–350 cells per μL, 351–500 cells per μL, and >500 cells per μL), HIV viral load (≤400 copies per ml vs. >400 copies per ml), AIDS-defining illness (yes vs. no), ART use (prevalent user vs. new user), clinic type (village clinic vs. township or county hospital), and treatment regimen for index cases (first-line regimen vs. others), and also HCV infection for non-index partners (yes vs. no) and unprotected sex in the recent year among couples (yes vs. no).

To further understand the independent effect of HCV infection and its joint effect with unprotected sex on HIV acquisition, we divided participants into four groups based on HCV infection at baseline (yes or no) and unprotected sex (yes or no). The cumulative risk of HIV seroconversion was compared between the four groups using a log-rank test. Cox proportional hazards regression model was conducted to determine the hazards of HIV seroconversion over 7 years of follow-up and reported as a relative risk estimate. Univariate and multivariate analyses were respectively examined. The joint impact of unprotected sex between couples (yes or no) and HCV infection of non-index partners (yes or no) during follow-up was also examined using the Cox proportional hazards regression model. Hazard ratios (HRs) and their 95% confidence intervals (CIs) were reported.

We examined the combined impact of unprotected sex and HCV infection on HIV seroconversion on the additive scale by relative excess risk due to interaction (RERI), attributable proportion (AP), and synergy index (SI) and their confidence intervals (20). RERI is an estimate of excess risk that is attributable to the interaction between the two exposures. AP is defined as the proportion of risk that is attributable to the interaction between unprotected sex and HCV infection. SI is a ratio that estimates whether a synergistic (SI > 1) or antagonistic (SI < 1) interaction exists between two exposures. Confidence intervals of three interaction measures were based on the Delta method described by Hosmer and Lemeshow (21, 22).

All analysis processes were performed using SPSS version 19.0 (IBM Corp. Released 2010. IBM SPSS Statistics for Windows, Version 19.0. Armonk, NY: IBM Corp) and SAS version 9.4 (SAS Institute Inc. 2013. Base SAS® 9.4 Procedures Guide. Cary, NC: SAS Institute Inc.).

## Results

There were 4,196 couples with index cases receiving ART. Among them, 42.97% of index cases were men and their average age was 44.60 years. Most index cases were farmers who got HIV infection through former blood or plasma donation. At baseline, 75.07% of index cases had AIDS-defining illness; and 81.96% initiated ART before the study enrollment (named “prevalent user”), while others began ART use at the baseline visit (named “new user”). About 60% of cases received ART at village clinics and two-third were on standard first-line treatment regimens, including AZT + 3TC + NVP/EFV, D4T + 3TC + NVP/EFV, and TDF + 3TC + NVP/EFV (Table 1).

**Table 1:** Baseline characteristics of index partners receiving ART.

	**Total (%)**		**Total (%)**
Sex		CD4 count (cells per μL)^‡^	
Male	1803 (42.97)	<200	882 (21.36)
Female	2393 (57.03)	200–350	1205 (29.18)
Age (years)		351–500	956 (23.15)
<50	3152 (75.12)	>500	1087 (26.32)
≥50	1044 (24.88)	AIDS–defining illness^§^	
Education		Yes	3146 (75.07)
No schooling or primary school	2404 (57.29)	No	1045 (24.93)
Middle school and above	1792 (42.71)	ART use	
Occupation		Prevalent user	3439 (81.96)
Farmer	4022 (95.85)	New user	757 (18.04)
Non–farmer	174 (4.15)	Clinic type	
Infection route		Village clinic	2508 (59.77)
Blood or plasma donation	3239 (77.19)	Township or county hospital	1688 (40.23)
Blood transfusion	678 (16.16)	ART regimen^¶^	
Sexual contact	227 (5.41)	AZT+3TC+NVP/EFV	819 (20.31)
Others	52 (1.24)	D4T+3TC+NVP/EFV	1581 (39.20)
Viral load (copies per ml)^†^		TDF+3TC+NVP/EFV	148 (3.67)
≤ 400	2584 (64.49)	Other	1485 (36.82)
>400	1423 (35.51)		

† *missing data on baseline viral load for 189 out of 4,196 index cases*.

‡ *missing data on baseline CD4 cell count for 66 out of 4,196 index cases*.

§ *missing data on baseline AIDS syndrome for 5 out of 4,196 index cases*.

¶ *missing data on baseline treatment regimen for 163 out of 4,196 index cases*.

*AZT, zidovudine; 3TC, lamivudine; NVP, nevirapine; EFV, efavirenz; D4T, stavudine; TDF, tenofovir*.

**Figure 2:**
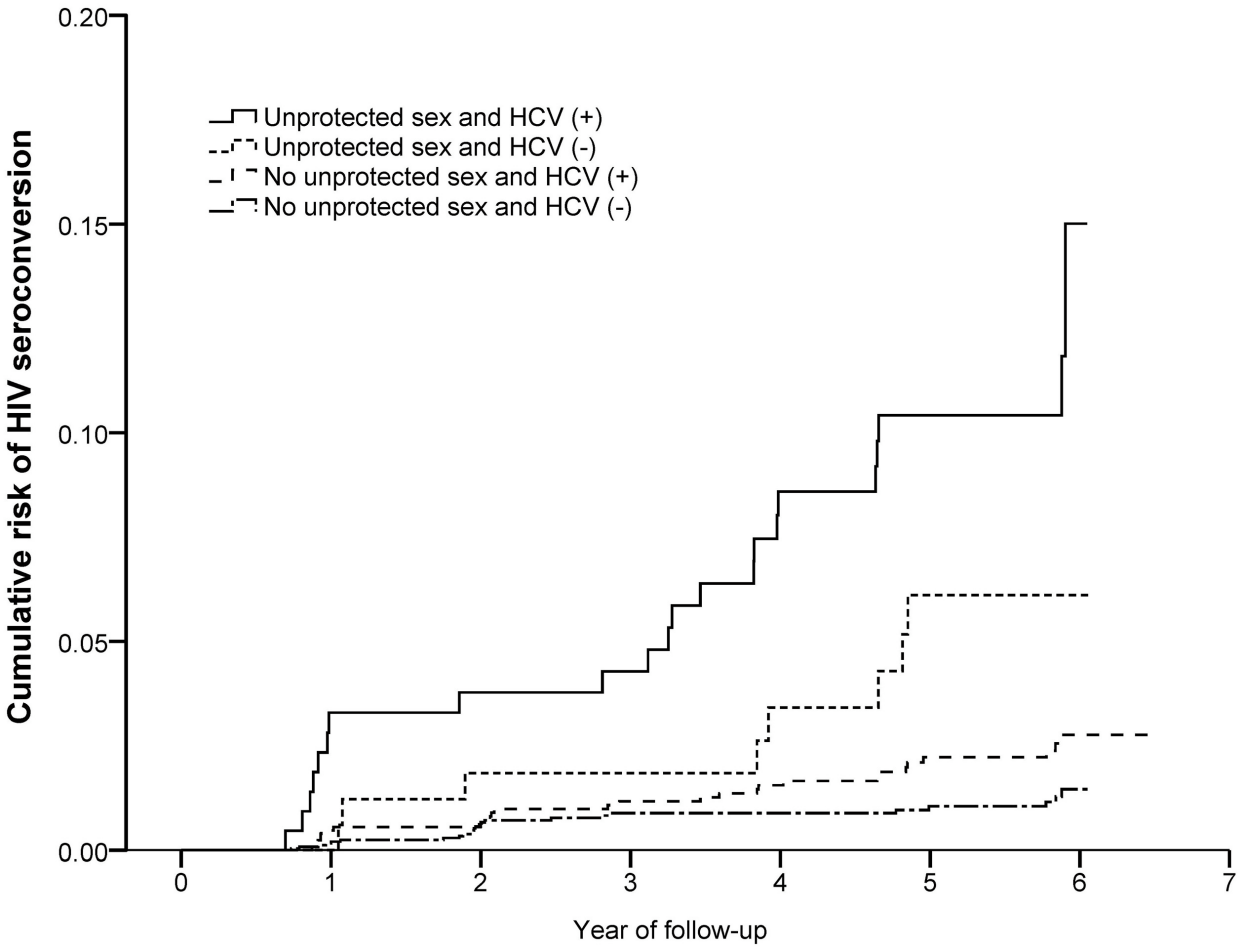
The joint effect of unprotected sex and HCV infection for HIV seroconversion of non-index partners among HIV serodiscordant couples with index cases receiving ART. Shown are Kaplan–Meier curves for HIV seroconversion progress in four groups based on marital unprotected sex and HCV infection of non-index partners.

There were 18,370.39 PY of follow-up and 81 partners had HIV seroconversions over the study period, with the overall rate of HIV seroconversion 0.44 per 100 PY (95% CI: 0.43–0.45). Couples, whose index cases were aged 50 years and above, had a baseline viral load >400 copies per ml and no AIDS-defining illness, and newly-initiated ART in the study period had a higher risk of HIV seroconversion. The interaction between unprotected sex in the recent year among couples and HCV infection of non-index partners was found statistically significant in HIV seroconversion. According to the Kaplan–Meier estimate, the risk of HIV seroconversion was examined in four groups stratified by unprotected sex in the recent year among couples and HCV infection of non-index partners (Figure 2).

The cumulative risk of HIV seroconversion was highest in couples with both unprotected sex and HCV (+) non-index partners, followed by those only having unprotected sex. The lowest risk was among couples with protected sex and HCV (–) non-index partners. For unprotected sex groups, the cumulative risk of HIV seroconversion was 4.71% and 10.19% among couples of HCV (–) and HCV (+) non-index partners. Among protected sex groups, the cumulative risk was 0.95 % and 2.11% among HCV (–) and HCV (+) non-index partners (Table 2).

**Table 2:** Numbers of HIV seroconversion of non-index partners and numbers of couples at risk by four groups.

	**HIV seroconversion**	**Couples at risk**	**%**
No unprotected sex and HCV (–)	24	2,532	0.95
No unprotected sex and HCV (+)	27	1,278	2.11
Unprotected sex and HCV (–)	8	170	4.71
Unprotected sex and HCV (+)	22	216	10.19

**Figure 3:**
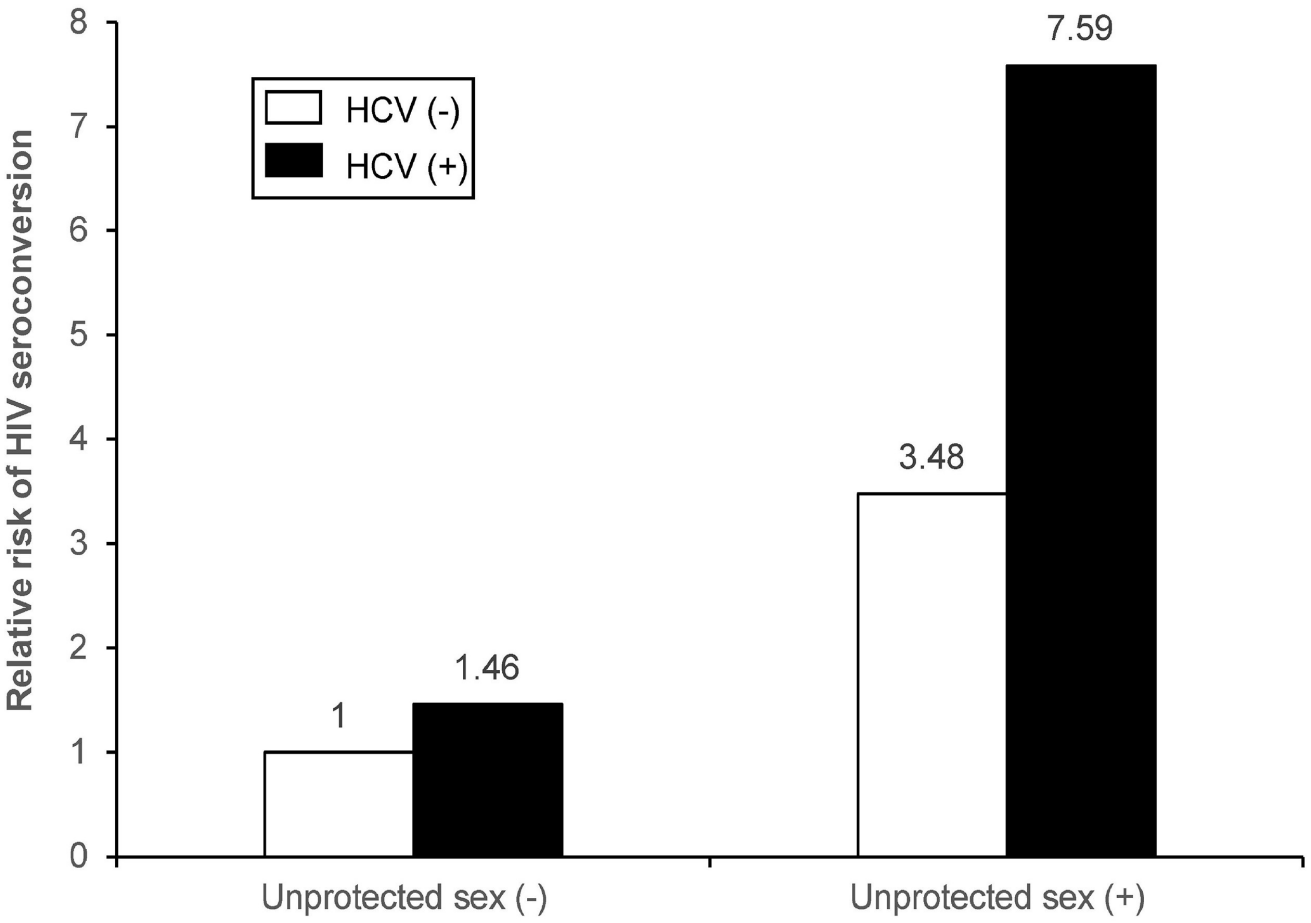
Joint effect and independent effects of unprotected sex and HCV infection on HIV seroconversion of non-index partners among HIV serodiscordant couples with index cases receiving ART in multivariate Cox proportional hazards regression model.

The combined effects of unprotected sex and HCV infection were statistically significant for the synergistic association with HIV seroconversion, with the highest risk among couples having both unprotected sex and HCV (+) non-index partners (HR=8.78; 95% CI: 4.92–15.67; *p* < 0.001), followed by couples with unprotected sex and HIV (–) non-index partners (HR=4.31; 95% CI:1.93–9.59; *p* < 0.001). The risk of HIV seroconversion was higher among couples only having HCV (+) than those with both protected sex and HCV (–) (HR=1.86; 95% CI:1.07–3.22; *p* < 0.001) (Figures 2, 3). The results remained consistent in multivariate analysis (Table 3).

**Table 3:** Univariate and multivariate analysis for HIV seroconversion of non-index partners among couples with index cases receiving ART.

**Variables**	**Seroconversion**	**PY**	**Seroconversion rate (per 100 PY)**	**Univariate**		**Multivariate**	
				**HR**	***P***	**aHR**	***P***
				**(95% CI)**		**(95% CI)**	
Sex of index cases							
Male	34	7753.27	0.44	1.00		1.00	
Female	47	10617.12	0.44	1.02 (0.65–1.58)	0.95	1.30 (0.79–2.13)	0.30
Age of index cases							
<50y	55	14100.93	0.39	1.00		1.00	
≥50y	26	4269.46	0.61	1.58 (0.99–2.51)	0.06	1.71 (1.01–2.89)	0.04
Education of index cases						
More than primary	29	7953.18	0.36	1.00		1.00	
Primary or less	52	10417.21	0.50	1.39 (0.88–2.19)	0.16	1.26 (0.74–2.15)	0.39
CD4 count (cells/μL) of index cases					
>350	31	9323.17	0.33	1.00		1.00	
≤ 350	47	8958.58	0.52	1.60 (1.02–2.52)	0.04	1.46 (0.89–2.41)	0.13
Viral load (copies/ml) of index cases				
≤ 400	33	11454.76	0.29	1.00		1.00	
>400	43	6538.59	0.66	2.29 (1.46–3.60)	<0.01	2.24 (1.35–3.70)	<0.01
AIDS–defining illness of index cases			
Yes	55	14928.47	0.37	1.00		1.00	
No	21	3437.14	0.61	1.75 (1.05–2.90)	0.03	1.80 (1.06–3.06)	0.03
ART use of index cases					
Prevalent users	68	16265.53	0.42	1.00		1.00	
New users	13	2104.86	0.62	1.58 (0.86–2.92)	0.14	2.41 (1.16–5.01)	0.02
Clinic type of index cases					
Higher or above	17	6974.72	0.24	1.00		1.00	
Village clinics	64	11395.67	0.56	2.31 (1.35–3.95)	<0.01	1.60 (0.83–3.10)	0.16
ART regimen of index cases					
First–line regimen	39	10386.02	0.38	1.00		1.00	
Others	39	7313.90	0.53	1.40 (0.90–2.19)	0.14	1.29 (0.77–2.17)	0.34
Unprotected sex/HCV status						
No/HCV (–)	24	10334.38	0.23	1.00		1.00	
No/HCV (+)	27	6184.18	0.44	1.86 (1.07–3.22)	0.03	1.46 (0.77–2.78)	0.25
Yes/HCV (–)	8	787.65	1.02	4.31 (1.93–9.59)	<0.01	3.48 (1.37–8.85)	<0.01
Yes/HCV (+)	22	1064.18	2.07	8.78 (4.92–15.67)	<0.01	7.59 (3.88–14.88)	<0.01

According to the measures of synergistic interaction between unprotected sex and HCV infection on risk of HIV seroconversion, we found that RERI, AP, and SI were 3.65 (95% CI: 0.61–6.70), 0.48 (95% CI: 0.18–0.79), and 2.24 (95% CI: 1.01–5.02), respectively. It indicated an additive interaction between unprotected sex among couples and HCV infection of non-index partners in their joint effect with HIV seroconversion risk.

In multivariate analysis, in addition to unprotected sex among couples and HCV infection of non-index partners, a higher risk of HIV seroconversion was also found among couples with older index partners (HR=1.71, 95% CI: 1.01–2.89), having higher viral load (HR=2.24, 95% CI: 1.35–3.70), no AIDS-defining illness (HR=1.80, 95% CI: 1.06–3.06), and new ART user (HR=2.41, 95% CI: 1.16–5.01).

## Discussion

Our finding suggested a relationship between HCV prior infection and the risk of HIV acquisition among heterosexual HIV serodiscordant couples with index cases receiving ART. We also evaluated the interaction between HCV infection and unprotected sex on HIV acquisition on the additive scale and multiplicative scale, showing that HCV infection might facilitate HIV acquisition especially if concurrently having unprotected sex.

It was identified that unprotected sex was an independent risk factor for HIV transmission among serodiscordant couples even with index partners on ART, strengthening the importance of safe sex behavior in HIV transmission. As is also known, condom use could not provide absolute protection against HIV even in situation when safety measures have been taken during sexual activity. Reasons might be attributed to incorrect and inconsistent condom use or poor condom quality (23).

In this study, non-index partners with HCV prior infection had a higher risk of HIV acquisition. It might be explained by the fact that HCV prior infection changed the normal immune response, increasing the susceptibility to HIV infection. HIV and HCV infections shared similar immunopathogenic mechanisms, as cellular immune responses are believed to be critical to the control of virus replication in both the viruses (24). For partners with prior HCV infection, their immune system would be activated. The activated state of immune response with a CD4 count increase might promote HIV infection and replication (25). For couples with unprotected sexual contact, the effect of HCV prior infection on HIV acquisition of non-index partners has been further amplified, thereby increasing the risk of HIV transmission among couples.

We also confirmed the previous evidence that patients aged old and having high-plasma HIV viral load might have a higher risk of HIV transmission (26, 27). Older couples were usually poorly educated and lack sufficient knowledge and awareness of HIV prevention. Besides, for patients with higher HIV viral load, only if the amount of HIV viral load in blood and other body fluids is successfully suppressed to an undetectable level through ART, the risk of HIV transmission could be greatly reduced. In a modeling study, with a decrease in average plasma HIV-1 RNA of 0.74 log10 copies/ml, the risk of heterosexual transmission could be reduced by 50% (28). Index cases newly initiating ART at baseline had a higher risk of HIV transmission than prevalent users, demonstrating the heterogeneity of outcome occurrence due to the impact of cumulative exposure risk among patients at different timing of treatment initiation which is common in pharmaceutical epidemiology (29).

It was worth noting that the risk of HIV seroconversion was significantly higher among couples of index cases without AIDS-defining illness at baseline. This demonstrated that for couples, whose index cases were asymptomatic and receiving treatment, unprotected sex might occur. It has been reported that the preventive effect of treatment could be offset by risk compensation behaviors of HIV infections when prevention technologies are used (30, 31). Therefore, in the case of partially effective biomedical technology, special attention should be paid to the behavioral changes that may be induced by subjects receiving intervention measures.

There are some limitations that should be noted. First, HIV seroconversion observed represented HIV transmission between couples, since HIV index cases in this population were largely older patients with low rates of drug use, casual sex, and homosexual behaviors (23). Second, the information on condom use and sexual behavior was self-reported. We cannot possibly rule out the overestimation of condom use reported by participants themselves. However, the extent of condom protection was always difficult to quantify because of many methodological challenges inherent in studying privacy behavior that cannot be directly observed or measured (32, 33). Finally, this study was based on the secondary database which did not collect HCV status of index cases and other risk factors like STIs or genital ulcers. Further studies should be carried out to validate the causal relationship between HCV infection on HIV acquisition and the interaction effect of HCV and unprotected sex.

## Conclusion

This study not only showed the effect of HCV infection on the risk of HIV acquisition but also provided an epidemiological basis for the synergistic effect of HCV infection and unprotected sex on HIV sexual transmission between couples. Recent studies have suggested that HIV infection is rising in China and worldwide. Given the large burden of the HIV and HCV epidemic, we believe that our findings may have important public health significance, especially in countries with a higher prevalence of HCV. As HCV infection is not only an independent risk factor but also a probable modifiable risk factor of HIV acquisition, HCV screening should be tested among HIV high-risk population. Further studies should be performed to refine the understanding of the biological mechanism of HCV prior infection and subsequent HIV acquisition.

## Data Availability Statement

The original contributions presented in the study are included in the article/supplementary material, further inquiries can be directed to the corresponding authors.

## Ethics Statement

This study was approved by the institutional review board of the National Center for AIDS/STD Control and Prevention at the Chinese Center for Disease Control and Prevention (IRB:00002276). All study participants provided written informed consent. The procedures performed involving study participants were in accordance with the ethical standards of the institutional and/or national research committee and with the 1964 Helsinki declaration and its later amendments or comparable ethical standards.

## Author Contributions

FC, LaW, and LuW conceived and drafted the article. FC, HT, JH, DL, and PL verified, compiled, and analyzed data. NW, MH, LaW, and LuW made critical revisions to the article. All authors contributed to the interpretation of results, manuscript revisions, and read and approved the final manuscript.

## Conflict of Interest

The authors declare that the research was conducted in the absence of any commercial or financial relationships that could be construed as a potential conflict of interest.

## Publisher's Note

All claims expressed in this article are solely those of the authors and do not necessarily represent those of their affiliated organizations, or those of the publisher, the editors and the reviewers. Any product that may be evaluated in this article, or claim that may be made by its manufacturer, is not guaranteed or endorsed by the publisher.
